# Doses for experiments with microbeams and microcrystals: Monte Carlo simulations in RADDOSE‐3D

**DOI:** 10.1002/pro.3922

**Published:** 2020-08-18

**Authors:** Joshua L. Dickerson, Elspeth F. Garman

**Affiliations:** ^1^ Department of Biochemistry University of Oxford Oxford United Kingdom

**Keywords:** dose, microbeams, microcrystals, photoelectron escape, RADDOSE‐3D, radiation damage

## Abstract

Increasingly, microbeams and microcrystals are being used for macromolecular crystallography (MX) experiments at synchrotrons. However, radiation damage remains a major concern since it is a fundamental limiting factor affecting the success of macromolecular structure determination. The rate of radiation damage at cryotemperatures is known to be proportional to the absorbed dose, so to optimize experimental outcomes, accurate dose calculations are required which take into account the physics of the interactions of the crystal constituents. The program RADDOSE‐3D estimates the dose absorbed by samples during MX data collection at synchrotron sources, allowing direct comparison of radiation damage between experiments carried out with different samples and beam parameters. This has aided the study of MX radiation damage and enabled prediction of approximately when it will manifest in diffraction patterns so it can potentially be avoided. However, the probability of photoelectron escape from the sample and entry from the surrounding material has not previously been included in RADDOSE‐3D, leading to potentially inaccurate does estimates for experiments using microbeams or microcrystals. We present an extension to RADDOSE‐3D which performs Monte Carlo simulations of a rotating crystal during MX data collection, taking into account the redistribution of photoelectrons produced both in the sample and the material surrounding the crystal. As well as providing more accurate dose estimates, the Monte Carlo simulations highlight the importance of the size and composition of the surrounding material on the dose and thus the rate of radiation damage to the sample. Minimizing irradiation of the surrounding material or removing it almost completely will be key to extending the lifetime of microcrystals and enhancing the potential benefits of using higher incident X‐ray energies.

## 1 INTRODUCTION

Radiation damage, which has been a major concern in MX for over 50 years,^1^ is caused as X‐rays deposit energy in the sample as they interact with it through the photoelectric and Compton effects. This limits the amount of signal that can be obtained before the structural integrity of the sample is severely compromised (crystal lifetime). The use of cryogenic temperatures of ~100 K reduces radiation damage rates by a factor of ~30–70 compared to that at room temperature.^2^, ^3^ However, the development of third and fourth generation synchrotrons with higher flux density X‐ray beams has brought the issue of radiation damage back into sharp focus. For an overview of the issues surrounding radiation damage in MX, the reader is directed to reviews by Holton^4^ and Garman and Weik.^5^, ^6^

A widely used metric that has been useful in providing a reproducible “*x*‐axis” against which to plot various damage indicators and thus monitor the effects of radiation damage is the absorbed dose, defined as the energy absorbed per unit mass in SI units of gray (Gy = J/kg). Consideration of the absorbed dose allows MX experiments to be planned so the sample dose does not exceed a dose limit at which the integrity of the sample is severely compromised.^7^, ^8^ The dose cannot be measured directly but must be estimated using knowledge of the cross sections for the relevant interactions and the relevant experimental parameters: crystal (size and composition), beam (intensity profile, energy, flux, size), and exposure time.

Such calculations show that when a 100 μm sized crystal containing no heavy atoms is irradiated by 12.4 keV (≡1 Å) X‐rays, only ~2% of the incident beam will interact with the crystal in any way.^9^ Of this ~2%, only ~8% will scatter elastically (Thomson scattering) and contribute productively to the diffraction pattern. Another ~8% of the interacting X‐rays will interact by the Compton effect (inelastic scattering), during which a photon loses some energy to a recoil electron, with the total momentum of the system being conserved. However, the major interaction at this X‐ray energy is the photoelectric effect, in which the photon is totally absorbed and a photoelectron, usually from the K shell of the atom, is released with energy equal to the photon energy minus the electron shell binding energy. The ion produced by the emission of a photoelectron from an inner shell will relax by de‐excitation of an electron from a higher shell to fill the vacancy. This is then followed by the release of a fluorescent photon with energy equal to the electron transition energy or an Auger electron, with the probability of fluorescent emission increasing with increasing atomic number.^10^

**FIGURE 1:**
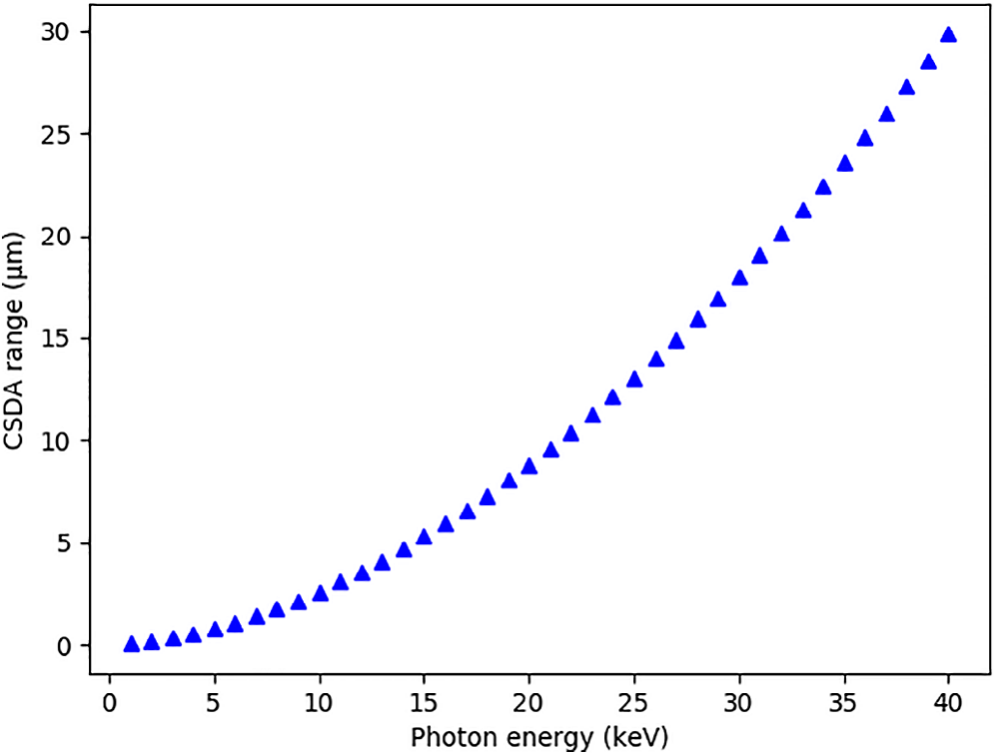
Range of electrons travelling through amorphous ice, calculated using the CSDA

In terms of deleterious effects, the photoelectrons are particularly damaging, with a 12 keV photoelectron potentially giving rise to ~500 further ionizations.^11^ When these electrons are produced within the sample, they have a finite chance of escaping before they have lost all of their energy, thus reducing the dose. Several papers have considered the effects of photoelectron escape on radiation damage rates in MX. Nave and Hill^12^ predicted that photoelectron escape would cause a significant radiation damage rate reduction in microcrystals by using the Monte Carlo program CASINO (monte CArlo SImulation of electroN trajectory in sOlids),^13^ which simulates a beam of electrons at a given starting energy moving through a user defined material. Intrinsic to these simulations is knowledge of the stopping power of the electrons (the average rate of energy loss per unit path length), and from this the range of the particle can be calculated using the continuous slowing‐down approximation (CSDA), as shown in Figure 1. The Nave and Hill simulations also suggested that the effect of photoelectron escape is more significant at higher incident X‐ray energies (*E*_*inc*_), and a similar method was employed by Cowan and Nave^14^ to suggest a significant advantage in collecting data at *E*_*inc*_ higher than the ~12.4 keV routinely used (20–30 keV *E*_*inc*_ was predicted to be optimum) for small crystals, providing other experimental factors remain optimized (e.g., detector efficiency etc.). More recent Monte Carlo simulations^15^, ^16^, ^17^ have arrived at similar conclusions, and the simulations from Dickerson *et al*. predicted a significant improvement on increasing incident energy from 12.4 to 26 keV for crystals of 5 μm or smaller, provided that a detector efficient at these higher energies is used.^17^ Experimentally, Sanishvili *et al*.^18^ demonstrated reduced radiation damage rates in protein crystals using micron‐sized 15.1 keV and 18.5 keV X‐ray beams and reconciled these results with Monte Carlo simulations to demonstrate the extent of the escape of photoelectrons from the diffracting volume of the crystal. For an 18.5 keV X‐ray beam, they observed damage at a distance of ~4 μm from the beam. Similarly, Finfrock *et al*.^19^ observed reduced radiation damage rates in protein crystals when using a submicrometre line‐focus beam, and observed damage at a distance of ~5 μm from an 18.6 keV X‐ray beam.

To allow the experiments mentioned above to be intercomparable, the absorbed dose must be calculated and specified. A convenient and freely available tool used by many experimenters to estimate doses over the last 16 years has been the RADDOSE and RADDOSE‐3D series of programs, which have been evolving and becoming more sophisticated with time. RADDOSE v1^20^ estimated the dose absorbed by the sample solely from the photoelectric effect. This went through several iterations, with RADDOSE v2^21^ including the possibility of the escape from the sample of fluorescent photons produced by atom relaxation following the emission of a photoelectron, a phenomenon that slightly reduces the dose. Subsequently RADDOSE v3 included the energy loss due to the Compton effect, which starts to become appreciable only at *E*_*inc*_ above 20 keV. These three RADDOSE versions were written in Fortran and were designed for a scenario in which the beam was bigger than the crystal, thus totally bathing it for the whole duration of the data collection.

However once X‐ray beams were routinely smaller than the crystals, this approach was no longer appropriate, and the current software, RADDOSE‐3D,^14^, ^15^ addresses this issue. RADDOSE‐3D divides the crystal into evenly spaced volume elements called voxels as well as dividing the crystal rotation into a discrete number of steps. This allows temporally and spatially resolved dose maps to be calculated as the crystal is irradiated and rotated, meaning that doses could now be estimated for beams smaller than the crystal.

Earlier versions of RADDOSE and RADDOSE‐3D had assumed that photoelectrons and Compton recoil electrons lose all of their energy where they are produced, until the recent incorporation of photoelectron escape into RADDOSE‐3D.^22^ To include this escape possibility, the linear path length of photoelectrons was determined analytically using a Gumbel distribution following extensive simulations using the program CASINO^23^ and photoelectrons were simulated travelling along linear tracks emanating in all directions from each voxel. However, this initial approach did not consider the full trajectory of the photoelectron, and additionally the effect of beam polarization on the preferential emission direction of photoelectrons was not included in the calculations. The additional dose due to the Compton effect was included, but the subsequently produced recoil electrons were assumed to lose their energy in the voxel in which they originated, rather than being tracked to their final destination. Most importantly, entry of photoelectrons from the material surrounding the crystal was not considered, and hence the calculations were only valid if this was assumed to be negligible.

This paper reports an extension of RADDOSE‐3D to perform full Monte Carlo simulations of a rotating crystal during MX experiments, which comprehensibly track the path and energy loss of photoelectrons and Compton recoil electrons produced both in the crystal and the surrounding material. This version (version 4), which includes an option for estimating time‐resolved doses for experiments using femtosecond pulses at Free Electron Lasers^24^ supersedes previous releases of the software. The simulations are presented, including an investigation of the conditions under which these provide more accurate dose estimates than previous versions of RADDOSE‐3D (v1–v3). Additionally, the effect that the material surrounding the crystal and sample orientation have on the dose is predicted.

## 2 DESCRIPTION OF THE PROGRAM

RADDOSE‐3D is an open source Java program that provides a full three‐dimensional representation of an MX experiment. The program divides the crystal into voxels and simulates its interaction with the X‐ray beam via a strategy described by the user (e.g., rotation and translation or both). The user describes a single crystal in the input and the program can simulate several data collection strategies via the wedge input block, with each wedge being associated with the most recently defined beam. A more complete description of the RADDOSE‐3D input is given by Zeldin *et al*.^25^ If the beam is smaller than the crystal, it is particularly important that the user specifies a large enough number of pixels (voxels) per micron to ensure that there are multiple voxels in each dimension.

### 2.1 *Program inputs*

**FIGURE 2:**
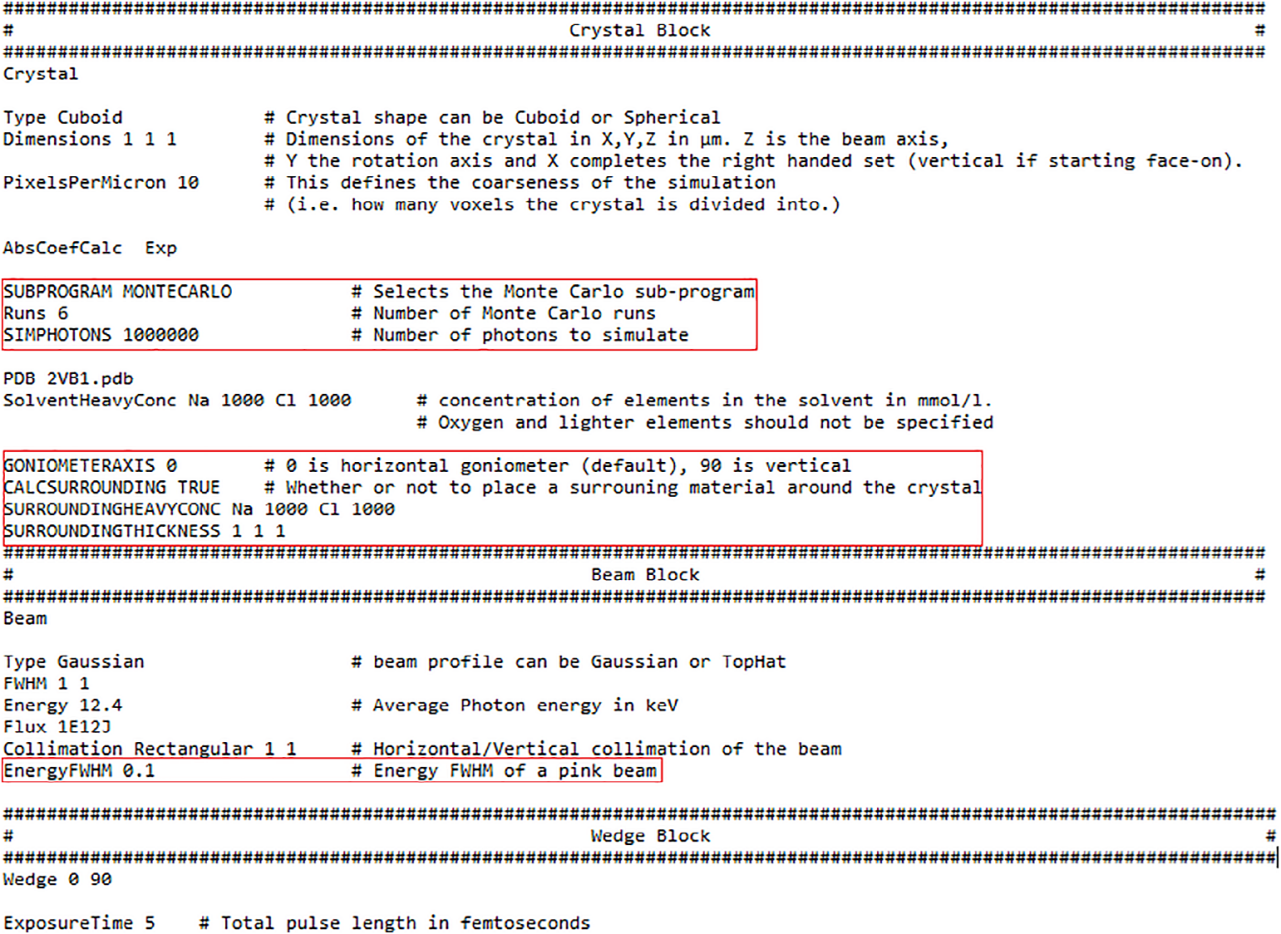
An example input file for the Monte Carlo simulations in RADDOSE‐3D. The extra inputs from a standard RADDOSE‐3D input file are highlighted in red

The input for the Monte Carlo simulation is the same as that of RADDOSE‐3D with a few extra specifiers, as described below and shown in Figure 2. In the “crystal” block, the user directs the program to the Monte Carlo simulations by specifying the subprogram as “MONTECARLO.” The number of times the program is to be run and the number of photons to be simulated per run (more photons is more accurate but increases runtime) must also be input as “runs” and “SIMPHOTONS,” respectively. It is recommended that the program is run multiple times to ensure the results are consistent. Still in the “crystal” block, the composition and size of the material surrounding the crystal can be defined. If no size is specified but “CALCSURROUNDING” is set to “true,” the thickness of the surrounding material is assumed to be the size of the maximum photoelectron range as calculated by the CSDA. If no composition of the surrounding material is specified, it is assumed to be pure water. If the surrounding material is not solution based (prior to cryocooling), the input “DENSITYBASED true” can be used and the elemental composition can then be defined with “SURROUNDINGELEMENTS” and the density with “SURROUNDINGDENSITY” (Figure S1). The rotation axis of the goniometer, with “0” being horizontal (which is the default if no axis is specified) and “90” being horizontal, can also be input.

In the “beam” block, a polychromatic beam can be defined by specifying the full width half maximum (FWHM) of the energy spread of the beam.

### 2.2 *Description of the code*

**FIGURE 3:**
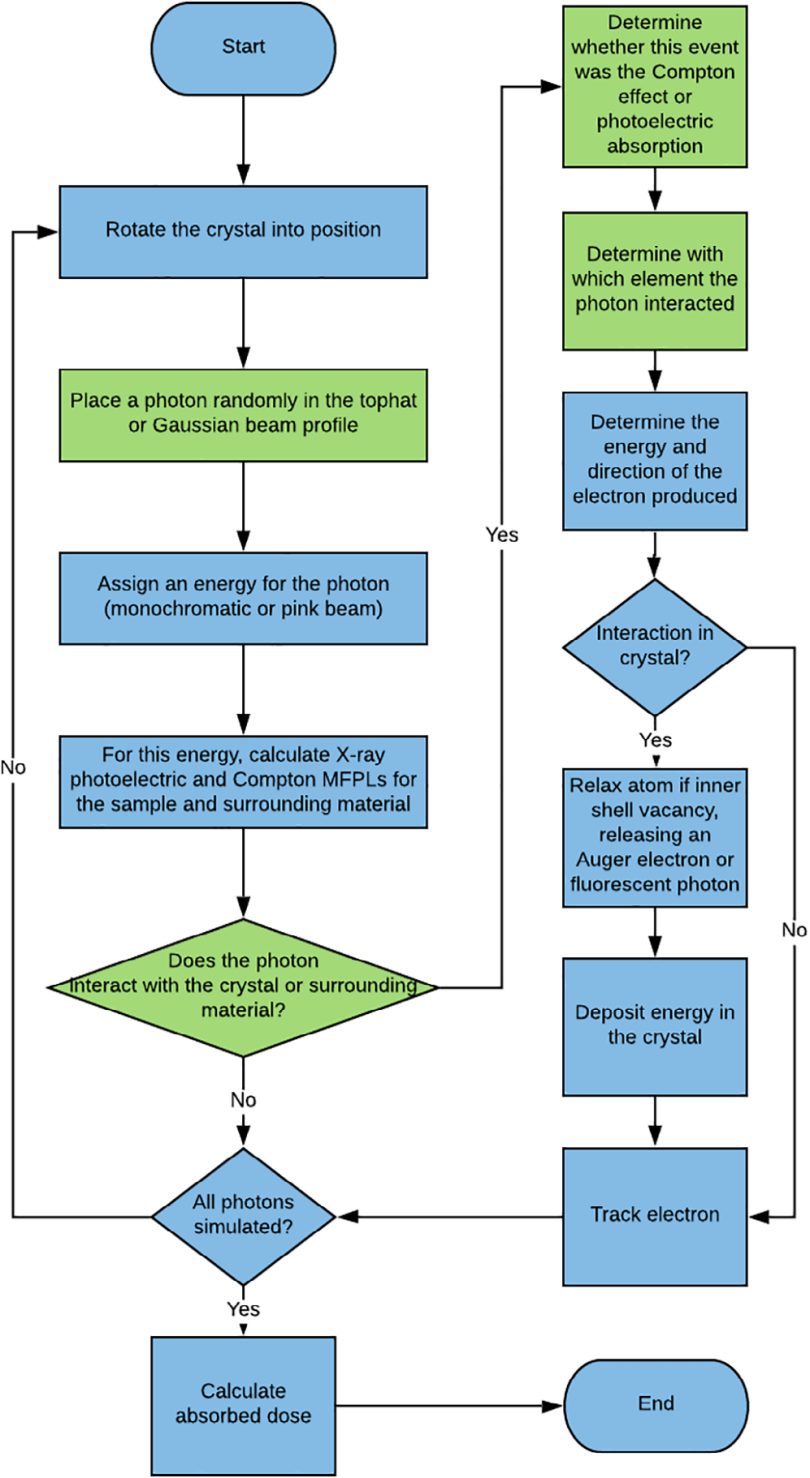
A description of the logical flow of the Monte Carlo simulations in RADDOSE‐3D. For green processes, random numbers were used to sample from probability density functions (PDFs)

For the Monte Carlo simulations (Figure 3), each photon to be simulated is tracked sequentially. The crystal and the surrounding material are first translated and rotated into position, with the *y‐*axis corresponding to the rotation axis of the goniometer, the *z‐*axis corresponding to the beam direction, and the *x‐*axis being the remaining axis. The *x‐* and *y‐*coordinates of the photon are assigned within the tophat or Gaussian beam profile by random inverse transform sampling, where a random number *u* between 0 and 1 is chosen and the largest value on the *x*‐axis of a cumulative distribution function such that *P(x)* ≤ *u* is returned. The photon is placed in front of the crystal, with the *z*‐coordinate corresponding to the start of the surrounding material (or crystal if no surrounding material is present). The energy of the photon is then assigned, which is based on systematic inverse transform sampling of a Gaussian distribution if a polychromatic beam is defined, or it is given the user specified energy if no beam energy FWHM is defined.

For the composition of the material currently traversed by the photon, the mean free path lengths (MFPLs) until the next ionizing interaction are then computed for the photoelectric and Compton effects, as well as for the total (*λ*_*photo*_, *λ*_*comp*_, and *λ*_*tot*_, respectively) by taking the inverse of the sum of all the atomic cross sections, $\sigma_{j}^{x}$, in the crystal, as shown in Equation (1):

$$\lambda_{\text{photo}}={\left[\sum_{j}\frac{\sigma_{j}^{\text{photo}}N_{j}}{V}\right]}^{-1}$$

$$\lambda_{\text{comp}}={\left[\sum_{j}\frac{\sigma_{j}^{\text{comp}}N_{j}}{V}\right]}^{-1}$$

(1) $$\lambda_{\mathrm{tot}}={\left[\frac{1}{\lambda_{\text{photo}}}+\frac{1}{\lambda_{\text{comp}}}\right]}^{-1}$$

where *N*_*j*_ is the number of atoms of element *j* in the unit cell of the crystal, denoted as the volume *V*. For each element *j*, the XCOM database^26^ is utilised to obtain the relevant scattering cross sections ($\sigma_{j}^{\text{photo}}$ and $\sigma_{j}^{\text{comp}}$).^26^

Using Equation (2),^27^ the distance to the next interaction, *s*, is then sampled^27^:

(2) $$s=-\lambda_{\mathrm{tot}}\ln\left(\mathrm{RND}\right)$$

where *RND* is a random number between 0 and 1.

For a photon interacting with either the crystal or the surrounding material, random inverse transform sampling is used to determine the type of interaction (Compton or photoelectric), according to the relative probabilities for the occurrence of each type of event. If this is photoelectric absorption, the initial photoelectron energy is set to *E*_*inc*_ minus the shell binding energy, with the inverse transform method again being used to calculate the elemental shell from which the photoelectron will originate, using the relative absorption probabilities for the different shells. If there is a vacancy in an inner shell, it is relaxed, and in order to calculate the energy of the Auger electron or fluorescent photon produced, it is necessary to identify the specific transition that has occurred. This is randomly sampled using the inverse transform method, with all the transition probabilities and also the relative emission probabilities of an Auger electron or fluorescent photon being obtained from the EADL (Evaluated Atomic Data Library) database.^28^, ^29^ For an Auger electron, which has low energy and thus a short path length, all its energy is assumed to have been lost in the voxel in which it originated. However, the fluorescent photons are not tracked and are assumed to escape (crystal volume or surrounding material). The error arising from this assumption is negligible since firstly the probability of fluorescent release is insignificant for low Z elements and secondly, for microcrystals fluorescent photons have a high probability of escape since their attenuation lengths will be bigger than the crystal volume.

The beam polarization direction biases the distribution of the initial emission direction of the photoelectron so that it is not random. The horizontal polarization of synchrotron radiation (Section S1) results in the photoelectrons being preferentially emitted in the direction of the polarization vector (Section S1).^30^, ^31^, ^32^, ^33^ Using the coordinate system defined in Section 2.2 and Equation (1) from Section S2,^34^, ^35^, ^36^, ^37^ the angle to the polarization vector is calculated, with its cosine providing the ejected electron direction vector *X* value. The *Y* and *Z* direction vector values are chosen randomly, with the condition that the magnitude of the vector is always 1. The photoelectrons (and any other electrons that are produced) are tracked using a polar coordinate system, with the coordinate transformations being calculated using Equation (3):

(3) $$\text{Electron direction vector}=\left(\begin{array}{c} X\\ Y\\ Z \end{array}\right)=\left(\begin{array}{c} \text{sinθcosφ}\\ \text{sinθsinφ}\\ \text{cosθ} \end{array}\right)$$

where *θ* is the polar angle (0 ≤ *θ* ≤ *π*) and *φ* is the azimuthal angle (0 ≤ *φ* < 2*π*).

For a Compton scattering event, the recoil electron energy (*E*_*comp*_) and angular emission direction (*θ*_*comp*_) are computed using Equations (4) and (5), respectively:

(4) $$E_{\text{comp}}=\frac{E_{\mathrm{inc}}^{2}\left(1-\cos\theta_{\mathrm{pho}}\right)}{mc^{2}\left[1+\left(\frac{E_{\mathrm{inc}}\left(1-\cos\theta_{\mathrm{pho}}\right)}{mc^{2}}\right)\right]}$$

(5) $$\tan\theta_{\text{comp}}=\frac{1}{\tan\left(\frac{\theta_{\mathrm{pho}}}{2}\right)\left(1+\frac{E_{\mathrm{inc}}}{mc^{2}}\right)}$$

where *m* is the rest mass of an electron, *c* is the speed of light, and a random angle in radians is chosen for the angular deflection of the photon, *θ*_*pho*_, where 0 ≤ *θ*_*pho*_ < *π*. Having lost *E*_*comp*_ to the electron, the Compton scattered photon is no longer tracked. The error caused by this is negligible since the total (Compton effect + photoelectric) MFPL in pure water is 2.07 mm for a 10 keV photon, there is only a <0.5% chance of another interaction in 10 μm of material. In addition, for a 10 keV photon, the Compton effect MFPL in pure water is ~30 times higher than that of the photoelectric effect, so the probability of a Compton event in the first place is very low.

**FIGURE 4:**
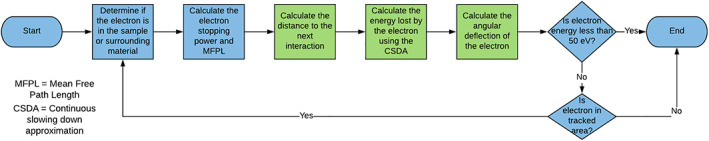
A description of the logical flow of how the electrons are tracked in the RADDOSE‐3D Monte Carlo simulations. For green processes, random numbers were used to sample from probability density functions (PDFs)

Once the initial direction of the photoelectron or Compton recoil electron has been chosen, the electron is tracked as in Figure 4. The electron elastic scattering cross sections are taken from tabulated values in the range 0.05–300 keV for each element, which were originally calculated using the program ELSEPA.^38^ The elastic scattering MFPL for the sample or surrounding material is then calculated as in Equation (1) and the distance to the next interaction, *s*, calculated from Equation (2).

The amount of energy lost by the electron over distance *s* is calculated using the continuous slowing down approximation (CSDA). This uses the collision stopping power which is the average energy loss per unit path length as a result of Coulomb collisions with bound atomic electrons,^39^ and is calculated as described in Section S2. The angular deflection of the primary electron is chosen using inverse transform sampling from tabulated differential cross sections (DCSs) calculated by the ELSEPA program. The direction vector of the electron is then updated in the 3D polar coordinate system as in Equation (3). The electron stopping power and MFPL is updated after each interaction until it either escapes the crystal and surrounding material, or its energy drops below 50 eV, in which case the remaining energy is considered to be deposited in that voxel.

For every photon simulated, the crystal is assumed to be pristine (i.e., the photon and electron cross sections are not updated after ionizations occur). This means that the dose is likely to be slightly overestimated, as it is in standard RADDOSE‐3D calculations. However, models have shown that for an “average protein,” there is an average of one ionization per atom at a dose of ~400 MGy.^24^, ^40^ Since the experimental dose limit of 30 MGy for synchrotron studies is an order of magnitude below this,^8^ it is unlikely in practice that a significant number of atoms are ionized at any stage of the diffraction experiment, so the anticipated error due to this treatment is minimal.

After all photons have been simulated, the energy deposited in each voxel is divided by the ratio of the number of photons simulated compared with the number incident on the crystal in the actual experiment. The final dose absorbed by a voxel, *D*_*n*_, is calculated using the following equation:

(6) $$D_{n}=\frac{J_{n}}{V_{n}\rho }$$

where *J*_*n*_ is the energy deposited in voxel *n*, *V*_*n*_ is the voxel volume, and *ρ* is the sample density.

### 2.3 *Program output*

The Monte Carlo simulations in RADDOSE‐3D give several output dose metrics, including the average dose across all voxels in the crystal (average dose whole crystal, ADWC) and the average dose across all voxels that fall within the beam area (average dose exposed region, ADER). Also output is a “RADDOSE‐3D style” dose calculated analytically from the relevant equations, where all energy from the photoelectric and Compton effects remains in the sample. The Monte Carlo “RADDOSE‐3D style” dose is routinely compared to the dose output by default RADDOSE‐3D calculations (i.e., not opting to include the effects of photoelectron escape) which do not use Monte Carlo methods. If there is a significant difference, a warning is displayed and the user is advised to increase the number of photons that are being simulated in the Monte Carlo procedure. The average diffraction weighted dose (DWD)^41^ is not output for the Monte Carlo simulations. To be reliably calculated, DWD requires several orders of magnitude more simulated photons than do the other dose metrics, and hence it is recommended that researchers should use ADER in cases where the beam size is smaller than the crystals.

## 3 RESULTS

### 3.1 *The effect on dose if using microcrystals and microbeams*

A systematic analysis was performed to determine in what situations the RADDOSE‐3D Monte Carlo simulations should be used to calculate dose to provide a more accurate value than the default RADDOSE‐3D calculations.

**FIGURE 5:**
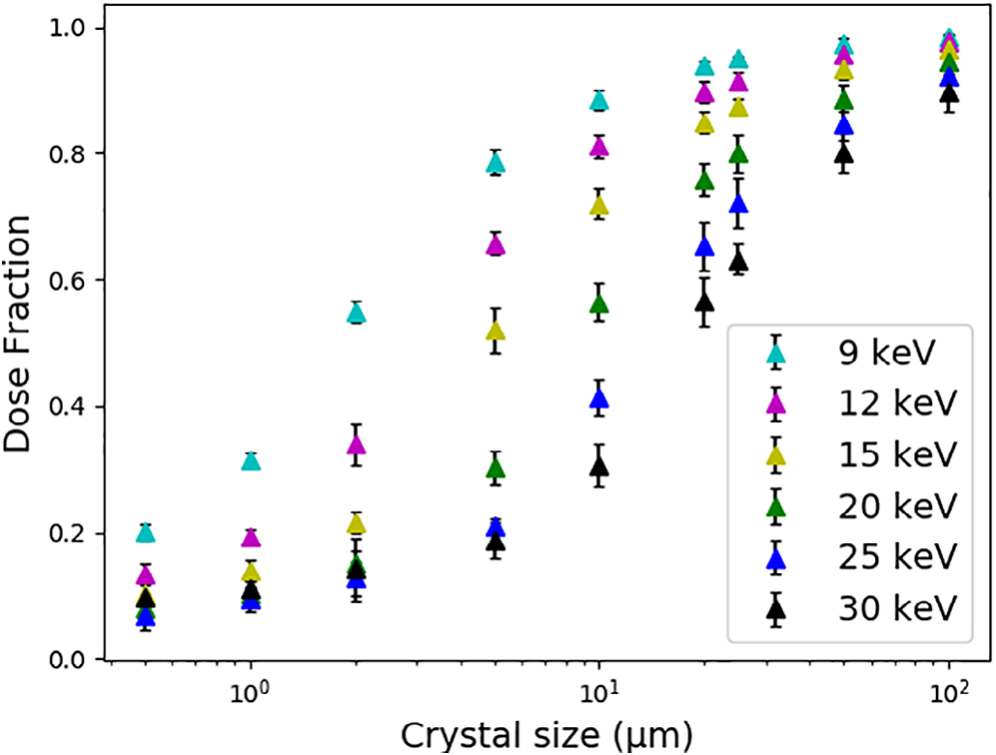
How the dose fraction (the ADER calculated by the Monte Carlo simulations divided by the equivalent “RADDOSE‐3D style” dose) varies with cubic crystal size for different *E*_*inc*_. In each case, the beam size is matched to the crystal size and no surrounding material is simulated. The rest of the input is as in Figure 2 (but with a monochromatic beam and no rotation) and error bars are 95% confidence limits with *n* = 6. Note the log scale on the *x*‐axis

The size of sample in which photoelectron escape from the crystal becomes significant was tested in the absence of surrounding material (Figure 5).

The larger the crystal, the lower the chance of photoelectron escape, with a greater than 20% reduction in dose for crystals smaller than 10 μm and an *E*_*inc*_ = 12 keV. The higher the beam energy, the greater the reduction in dose when including photoelectron escape, since the photoelectrons will have on average higher energy and hence travel further. For *E*_*inc*_ = 30 keV, there is a 20% reduction in dose for crystals smaller than 50 μm. Figure 5 also highlights the improvement in crystal lifetime from increased photoelectron escape that can be obtained by using a higher *E*_*inc*_, with an ~81% reduction in dose on simulating photoelectron escape for a 1 μm crystal at *E*_*inc*_ = 12 keV compared to a ~ 91% reduction in dose for *E*_*inc*_ = 20 keV compared with the no escape case. The potential advantages of using higher beam energies in MX for microcrystals is explored further in Dickerson and Garman.^17^

**FIGURE 6:**
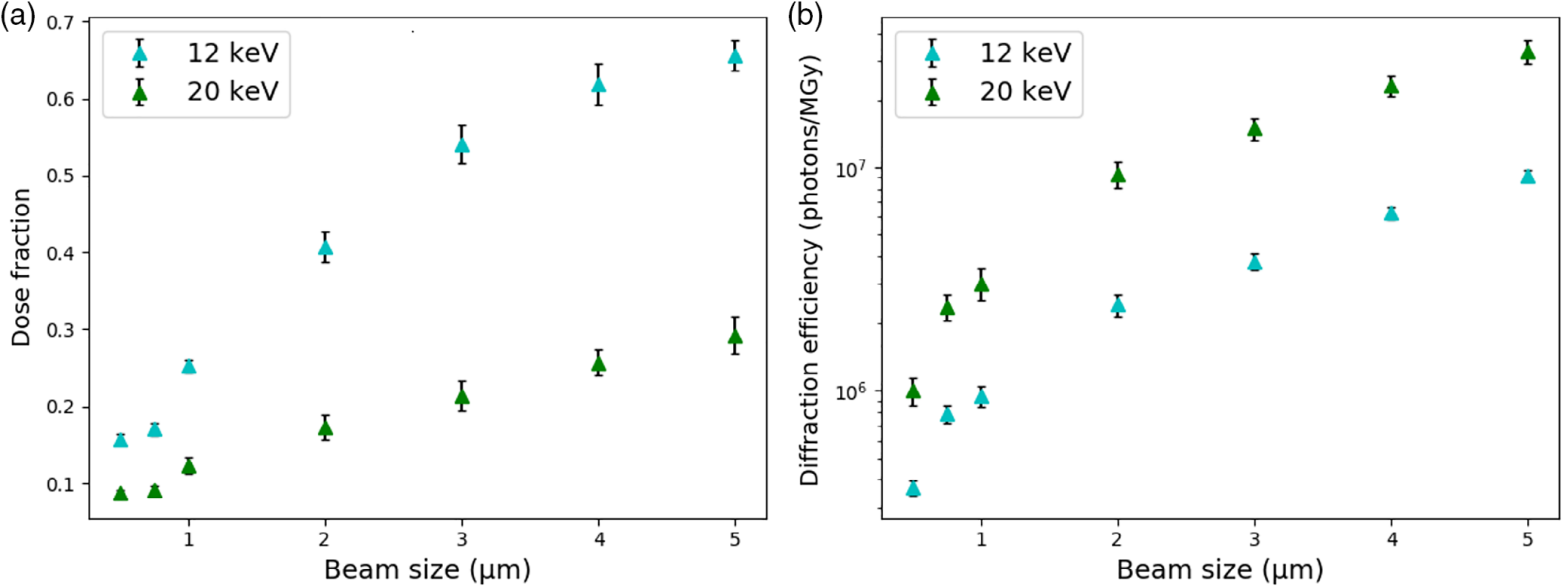
How the (a) dose fraction and (b) diffraction efficiency vary with beam size for different *E*_*inc*_. In each case, the crystal is a 5 μm cube and no surrounding is simulated. The rest of the input is as in Figure 2 (but with a monochromatic beam and no rotation) and error bars are 95% confidence limits with *n* = 6. Dose fraction is defined in the legend of Figure 5. Note the log scale on the *y*‐axis of (b)

A similar trend will be seen for microbeams that are smaller than the crystal, since photoelectrons will escape the irradiated volume. However, in spite of the reduction in dose for microbeams (Figure 6a), the diffraction efficiency (number of elastically scattered photons per MGy of absorbed dose) still increases since more of the sample is irradiated (Figure 6b).

### 3.2 *The pivotal importance of the surrounding material on the dose*

**FIGURE 7:**
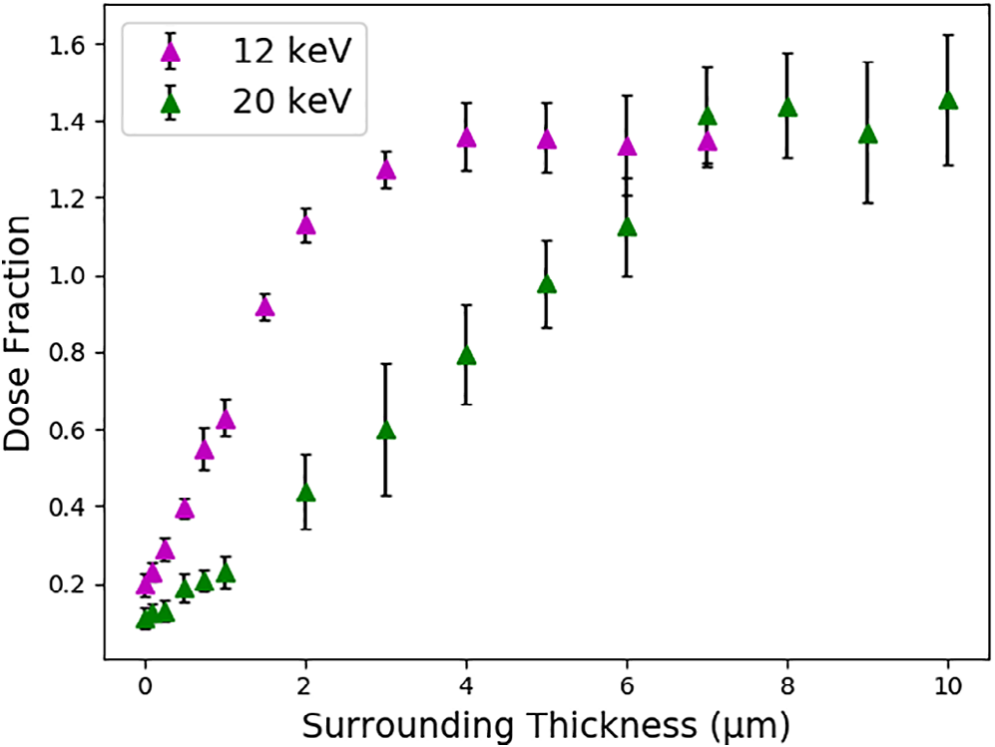
How the dose fraction varies with the thickness of the surrounding material for different *E*_*inc*_. In each case, the sample is a 1 μm cubic lysozyme crystal and the beam a rectangular tophat beam that fully illuminates the crystal and surrounding. The rest of the input is as in Figure 2 (but with a monochromatic beam and no rotation) and error bars are 95% confidence limits with *n* = 6

Although the results described in Section 3.1 show a large reduction in dose when the effect of photoelectron escape is included, when entry of photoelectrons and Compton recoil electrons from the surrounding material is also considered, the reduction in dose obtained from simulating electron escape can be completely removed, and there can even be an increase in dose since these photoelectrons can enter the crystal (Figure 7). The thickness and mM concentration of the amorphous ice based surrounding material can be user specified and simulations to investigate the effect on the ADER were carried out with increasing surrounding thicknesses. The dose increases approximately linearly and then begins to plateau as the surrounding thickness approaches the photoelectron range as calculated by the CSDA, which was 3.5 μm for *E*_*inc*_ = 12 keV, and 8.7 μm for *E*_*inc*_ = 20 keV (see Figure 1).

**FIGURE 8:**
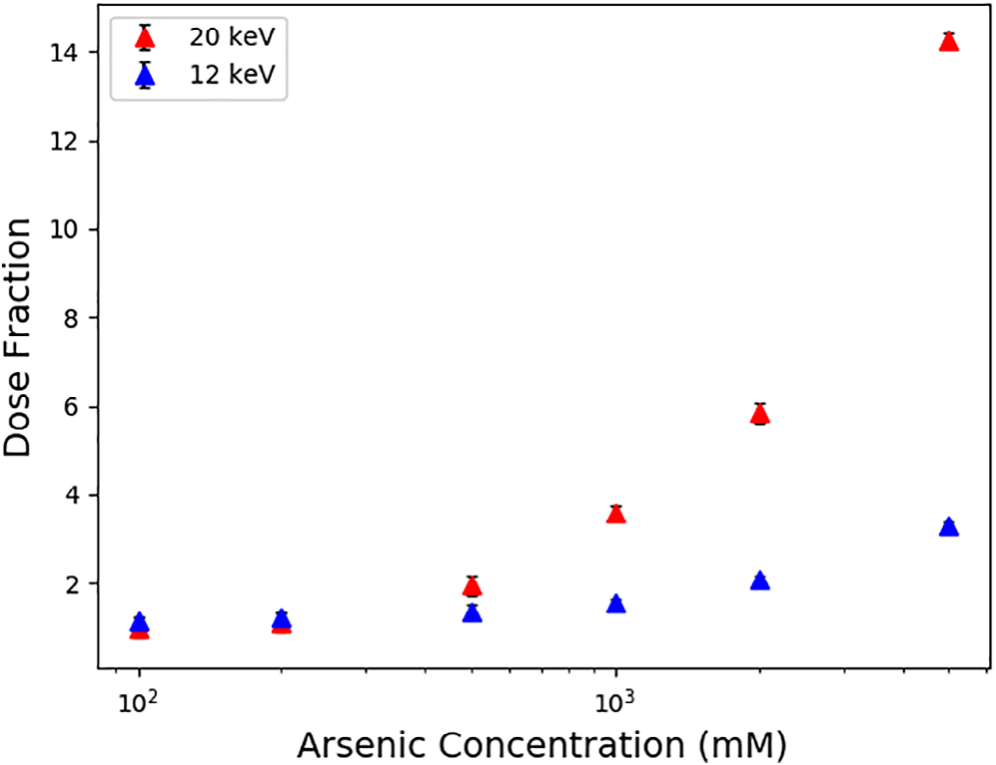
How the dose fraction varies with the composition of the surrounding material. The concentration of arsenic, which is a component of cacodylate containing buffers, is varied for a 1 μm cubic crystal and a 10 μm rectangular tophat beam. The rest of the input is as in Figure 2 (but with a monochromatic beam and no rotation) and error bars are 95% confidence limits with *n* = 6. The dose fraction is defined in the legend of Figure 5. Note the log scale on the *x*‐axis

As well as the thickness of the surrounding, it is also important that the composition of the surrounding is correctly defined. Heavier atoms have higher photoelectric cross sections and hence produce more photoelectrons. The heavier the atoms in the surrounding, the more photoelectrons will be produced and the higher the dose in the sample (Figure 8), as these photoelectrons can enter the crystal. This effect is particularly important if a beam much larger the crystal is used and when using higher *E*_*inc*_ incident X‐ray energies, since the volume from which photoelectrons can enter the crystal is increased (Figure 8).

In addition to specifying the surrounding material as a mM concentration of the constituents in amorphous ice, it can alternatively be defined based on its density and atomic elemental composition (Section S3). This feature can be used to define oil based cryoprotectants. As for the amorphous ice option, the number of photoelectrons produced will be dependent on the density of the oil and the size of the photoelectric cross sections of the constituent elements. A comparison of the dose calculated by RADDOSE‐3D Monte Carlo simulations for different oil based cryoprotectants (Table 1) shows that simulations of two of the three oil based cryoprotectants resulted in a lower dose than pure water, with polyphenyl ether and polyisobutylene having a ~25% and ~46% lower ADER, respectively. These results indicate that the most important factor when assessing the dose contributed by a surrounding material is the atomic number of its constituents, since photoelectric cross sections rise sharply with increasing atomic number.

**TABLE 1:** The changes in dose when different oils are simulated as a surrounding material.

Surrounding material	Composition	Density (g/cm^3^)	RADDOSE‐3D style ADER	Monte Carlo ADER
No surrounding	–	–	510.1 ± 36.5	95.7 ± 5.8
Water	H_2_O	1.00	524.5 ± 32.8	291 ± 25.1
Perfluoropolyether	C_2_F_6_(C_3_F_6_O)_*n*_	1.88	534.0 ± 52.1	572.9 ± 16.5
Polyisobutylene	(C_4_H_8_)_*n*_	0.92	525.0 ± 33.2	157.3 ± 5.9
Polyphenyl ether	(C_6_H_4_O)_*n*_	1.20	546.5 ± 39.7	217.6 ± 13.9

*Note*: The crystal was a 1 μm cubic crystal with a 2 μm rectangularly collimated tophat beam. The full input is shown in Figure S2. Polyisobutylene is the main component of Parabar 10312 (Paratone N) and the cryo oil Santovac® 5 is a 5 ring polyphenyl ether. The RADDOSE‐3D style ADER does not include the effects of photoelectron escape.

### 3.3 *Sample orientation*

To produce an accurate dose value, it may be important to specify the orientation of the crystal correctly. For example, Table 2 shows the effect of placing a rod‐shaped crystal in three different orientations.

**TABLE 2:** Effect of different crystal orientations on the dose calculated by the Monte Carlo simulations in RADDOSE‐3D

Orientation (μm)	RADDOSE‐3D style ADER (MGy)	Monte Carlo simulation ADER (MGy)	Dose fraction
*x* = 3, *y* = 1, *z* = 1	370.7 ± 20.5	76.3 ± 4.4	0.21 ± 0.02
*x* = 1, *y* = 3, *z* = 1	375.9 ± 10.0	120.0 ± 6.8	0.32 ± 0.02
*x* = 1, *y* = 1, *z* = 3	439.6 ± 20.0	98.8 ± 5.7	0.22 ± 0.02

*Note*: The crystal is rod‐shaped and the beam has a Gaussian intensity profile with FWHM 3 × 3 μm and *E*_*inc*_ = 12 keV. The rest of the input is as in Figure 1 and error bars are 95% confidence limits with *n* = 6.

In Table 2, the RADDOSE‐3D style ADER is higher when the long dimension is in the *z*‐axis. This is because a higher flux will be incident on this crystal since it is centered in the most intense part of the Gaussian beam. However, the Monte Carlo ADER is higher when the long dimension is in the *y*‐axis than when it is in the *x‐*axis. This is because the photoelectron emission direction is biased towards the beam polarization vector, which in this case of a horizontal goniometer on a synchrotron beamline is in the *y‐*axis direction. This means that fewer photoelectrons escape from the crystal when the long dimension is in the *y‐*axis, as indicated by an increased dose fraction for this orientation.

If the orientation of the crystal can be controlled, the consideration of electron escape will impact the optimal orientation of the crystal to maximize diffraction efficiency. This is illustrated in Table 3, where a plate‐like crystal with no surrounding material is irradiated by a large beam. In this case, the diffraction efficiency is highest when the small dimension is along the *y*‐axis, which is the beam polarization vector for a horizontal goniometer at a synchrotron beamline and hence there is greater photoelectron escape in this direction.

**TABLE 3:** The effect of different crystal orientations on the diffraction efficiency calculated from the Monte Carlo simulations in RADDOSE‐3D

Orientation (μm)	Dose fraction	Diffraction efficiency (photons/MGy)
*x* = 3, *y* = 3, *z* = 1	0.53 ± 0.03	8.56 × 10^5^ ± 1.14 × 10^5^
*x* = 3, *y* = 1, *z* = 3	0.27 ± 0.02	1.70 × 10^6^ ± 1.62 × 10^5^
*x* = 1, *y* = 3, *z* = 3	0.51 ± 0.04	7.88 × 10^5^ ± 1.21 × 10^5^

*Note*: The crystal is plate‐like and the beam has a tophat intensity profile with collimation 3 × 3 μm and *E*_*inc*_ = 12 keV. The wedge is a 360° rotation. The rest of the input is as in Figure 1 and error bars are 95% confidence limits with *n* = 6.

## 4 DISCUSSION

### 4.1 *When to use the Monte Carlo simulation option in RADDOSE‐3D*

Whether or not there is a significant difference between doses output by the Monte Carlo simulations and those from default RADDOSE‐3D estimates (no photoelectron escape included) will depend largely on: crystal size, beam size, beam energy, and the size and composition of the surrounding material. The smaller the size of the crystal or the beam, the bigger the difference between the dose values. This difference gets larger as beam energy increases, and at what crystal size this becomes significant for different beam energies can be obtained from the results shown in Figure 5.

However, the difference between the doses will be reduced from the values displayed in Figure 5 when the surrounding material is considered, since the dose calculated by the RADDOSE‐3D Monte Carlo simulations will continue to rise with increasing thickness of illuminated surrounding material up until the photoelectron range (Figure 7), as calculated by the CSDA. If this thickness of surrounding material is being illuminated, the Monte Carlo simulations are unlikely to produce a significantly different dose than the default RADDOSE‐3D (no photoelectron escape) calculations, unless the composition of the surrounding is significantly different to that of the crystal (Figure 8).

### 4.2 *The impact of the surrounding material*

As well as increasing the level of background, irradiation of the surrounding material can greatly increase the dose and thus the rate of radiation damage progression as a result of ionizations from photoelectrons produced in the surrounding. If a large enough volume of surrounding material is irradiated, the advantage of increased photoelectron escape when using higher *E*_*inc*_ will also be abolished. The increase in background and rate of radiation damage progression will be particularly detrimental to microcrystals, so it is important to reduce the negative effects of the surrounding material as much as possible.

If heavy atoms with a large photoelectric cross section are present in the mother liquor, backsoaking should be attempted to remove as many of these as possible to reduce the number of photoelectrons entering from the surroundings. An oil based cryoprotectant may also be beneficial, provided that it is composed of elements with a lower atomic number than oxygen. The next step would be to match the beam size to the crystal to ensure that the entire crystal is irradiated but as little surrounding material as possible is. However, the surrounding material in front and behind the crystal will still be irradiated, and also material around the sides of the crystal will be in the tails of the Gaussian intensity profile of the beam. It is most important to minimize irradiation of the surrounding material around the sides of the sample in the axis of the horizontal beam polarization vector, since photoelectron emission is biased along this axis so these photoelectrons will have the highest probability of entering the sample. If the beam size can be well matched to the crystal size, the increased photoelectron escape from using higher *E*_*inc*_ can also be realised, since the thickness of the irradiated surrounding material is more likely to be less than the photoelectron range in this case. There will thus be a greater number of photoelectrons escaping rather than entering the sample.

The ideal strategy to minimizing the negative effects of the surrounding material would be to reduce the thickness of it as much as possible without dehydrating the crystal. Sample grids have been designed to remove as much mother liquor as possible^42^, ^43^ and there has been some success using copper cryo‐EM grids^44^ for MX diffraction experiments. It has also been demonstrated that using layers of graphene sheets as a crystal mounting substrate can greatly the reduce the background scatter,^45^ which is also likely to substantially diminish the number of photoelectrons entering from the surrounding material since the graphene enclosing the crystal is incredibly thin at just 0.34 nm a layer.^30^, ^31^, ^32^, ^33^, ^34^, ^35^, ^36^, ^37^

### 4.3 *Orientation*

Given the large differences in calculated doses between different crystal orientations shown in Table 2, when the orientation of the crystal is unknown, which is likely to be the case in serial synchrotron crystallography, it is recommended that users average the MC simulation results over several or many orientations. For this, the wedge starting angle input can be used to step through the angle of the sample about the *y* axis, the “AngleP” input to alter the angle about the *z‐*axis, and “AngleL” to change the angle about the *x*‐axis. If the crystal is not necessarily centered in the middle of a Gaussian beam, the “startoffset” input can be used to translate the crystal off center.

As highlighted in Section 3.3, the beam polarization direction should be considered when determining which crystal orientation is optimal to maximize diffraction efficiency. However, many variables such as the beam profile and thickness of surrounding material will influence the optimal orientation. Thus, it is recommended that the RADDOSE‐3D Monte Carlo simulations are used prior to data collection to determine which orientation will give the highest diffraction efficiency.

### 4.4 *Effect of incident X‐ray energy*

As discussed in Dickerson *et al*.,^17^ using a higher incident X‐ray energy can improve the level of signal obtained before the crystal is significantly damaged (i.e., results in a higher diffraction efficiency), providing that a detector optimized for higher energy ranges is used. That study found an optimum *E*_*inc*_ of ~26 keV, with a decrease in diffraction efficiency occurring as *E*_*inc*_ is increased beyond this due to a drop in the detection efficiency of a CdTe detector and the increasing Compton scattering cross section. The present study explored the effect further, with a focus on the effect of the surrounding material. It is clear from the results presented in Figures 7 and 8 that irradiating a large volume of surrounding material reduces or eradicates the increased photoelectron escape seen at higher energies as a result of a greater volume of surrounding material from which photoelectrons can enter the crystal.

## 5 CONCLUSION

An extension to RADDOSE‐3D has been presented which allows users to perform Monte Carlo simulations that take into account the redistribution of photoelectrons and Compton recoil electrons from the crystal and surrounding material. This provides more accurate dose estimates for MX experiments conducted with microbeams and/or microcrystals than were previously available. The simulation results highlight the important role that the surrounding material can play in the dose absorbed by the crystal and thus in the rate of radiation damage progression. It is vitally important to reduce irradiation of the surrounding material to increase the diffraction lifetime of microcrystals in order to realise the benefits of higher incident X‐ray beam energies. Lastly, if orientation can be controlled, the Monte Carlo simulations can allow identification of the best crystal orientation to maximize diffraction efficiency. The program is freely available and can be found at https://github.com/GarmanGroup/RADDOSE-3D/releases.

In summary, the new RADDOSE‐3D MC simulation capability of the program allows researchers to estimate absorbed doses for their planned experimental parameters prior to the X‐ray irradiation of their crystals. Data collection conditions can then be adjusted in order to optimize the data quality when using microbeams and microcrystals.

## AUTHOR CONTRIBUTIONS

**Joshua Dickerson:** Conceptualization; investigation; methodology; software; writing‐original draft; writing‐review and editing. **Elspeth Garman:** Conceptualization; funding acquisition; investigation; project administration; software; supervision; writing‐original draft; writing‐review and editing.

## Supporting information


**Data S1** Supporting information
