# Supplementary material for: Doses for experiments with microbeams and microcrystals: Monte Carlo simulations in RADDOSE‐3D
Source: Protein Sci. 2020 Aug 18;30(1):8–19. doi: 10.1002/pro.3922 (PMC7737758; doi:10.1002/pro.3922)
Supplement: Supplementary file 1 — Data S1 Supporting information [file PRO-30-8-s001.docx]

**Doses for experiments with microbeams and microcrystals: Monte Carlo simulations in RADDOSE-3D**

*Joshua L. Dickerson and Elspeth F. Garman**

*Department of Biochemistry, University of Oxford, South Parks Road, Oxford, OX1 3QU*

**Email:* [*Elspeth.garman@bioch.ox.ac.uk*](mailto:Elspeth.garman@bioch.ox.ac.uk)

**Supplementary Information**

**Section S1: X-ray polarisation**

Since photoelectrons are preferentially emitted in the same direction as the polarisation vector of the X-ray beam ^30^ and synchrotron radiation is horizontally polarised ^31,,^ ^32^, this effect must be taken into account in computing the photoelectron angular distribution. The differential cross section for photoelectron emission, *dσ/dΩ*, is given by equation 1.

|  | $\frac{d\sigma}{d\Omega}=\left( \frac{\sigma_{total}}{4\pi} \right)\left[ 1+\beta P_{2}\left( cos\theta\right) \right]$ |  |
| --- | --- | --- |

where *σ* is the cross section*, Ω* is the solid angle, *σ_total_* is the total photoelectron cross section, $\beta$ is an asymmetry parameter, *P_2_(cos*$\theta$*) = 0.5(3cos^2^*$\theta$ *– 1)* and $\theta$ is the angle between the polarization vector and the direction of the ejected electron$.$

Depending on the particular element and the shell from which the photoelectron is ejected, the asymmetry parameter, $\beta$, varies between -1 and 2 and is always 2 for K shells. In the RADDOSE-3D simulations reported here, the emitted K-shell photoelectrons were polarised by using equation 1 to bias their track directions. The remaining electrons expected to emanate from other shells were left unpolarised. This treatment is likely to result in a slight underestimate of the degree of polarisation, but since K shells dominate the photoelectric cross section ^33^, this is a reasonable approximation.

**Section S2: Stopping powers**

For an atom, the collision stopping power (*S_col_*) can be calculated from equation 2 ^34,35^:

|  | $S_{col}=\rho\frac{2\pi N_{a}r_{e}mc^{2}}{\beta^{2}}\frac{Z}{A}\left( F\left( \beta\right)-\left( 2\ln I \right)-\delta\right)$ |  |
| --- | --- | --- |

with $F\left( \beta\right)$:

$$F\left( \beta\right)=\ln\left( \frac{mc^{2}E\beta^{2}}{2\left( 1-\beta^{2} \right)} \right)-\left( \left( 2\sqrt{1-\beta^{2}}-1+\beta^{2} \right)\ln2 \right)+1-\beta^{2}+\frac{1}{8}\left( 1-\sqrt{1-\beta^{2}} \right)$$

where *ρ* is the material density (kg/m^3^) *N_a_* is Avogadro’s number (6.022045$\times$10^23^ mol^-1^), *r_e_* is the classical electron radius (2.817940$\times$10^-15^ m), *m* is the electron rest mass (9.10956$\times$10^-31^ kg), *c* is the speed of light (2.99792458$\times$10^8^ m/s), *Z* and *A* are respectively the atomic number and atomic mass (g/mol) of the atom, , *δ* is the density effect correction (0 at the relevant energies and thus neglected ^36^ in the simulations reported here) and *E* is the kinetic energy of the scattered electron (J)*I* is the mean excitation energy which were taken from tabulated values in ICRU report 37 ^37^ and multiplied by 1.13 to modify them from being in the gas phase to the liquid/solid phase ^37^. $\beta$ = *v/c* where *v* is the velocity of the electron and $\beta$ can be obtained from equation 3:

|  | $\beta=\sqrt{1-\frac{1}{\gamma^{2}}}$ |  |
| --- | --- | --- |

$$\gamma=1+\frac{E}{mc^{2}}$$

The formalism above allows the collision stopping power for each constituent atom to be computed, but for the value for the entire sample is required. The sum Bragg additivity rule ^45^ states that for a compound the collision stopping power can be calculated by replacing the *Z/A* term in equation 2 above with the weighted sum of the atomic constituents:

|  | $\left\langle Z/A \right\rangle=\sum_{j} \omega_{j}\left( \frac{Z_{j}}{A_{j}} \right)$ |  |
| --- | --- | --- |

where the fraction of the total molecular weight in the unit cell that the *j^th^* atom contributes is *ω_j_* . In addition, this modifies the mean excitation energy, $I$:

|  | $\ln I=\left[ \sum_{j} \omega_{j}\left( \frac{Z_{j}}{A_{j}} \right)\ln I_{j} \right]\times\frac{1}{\left\langle Z/A \right\rangle}$ |  |
| --- | --- | --- |

**Section S3: Example of surrounding material defined as oil**


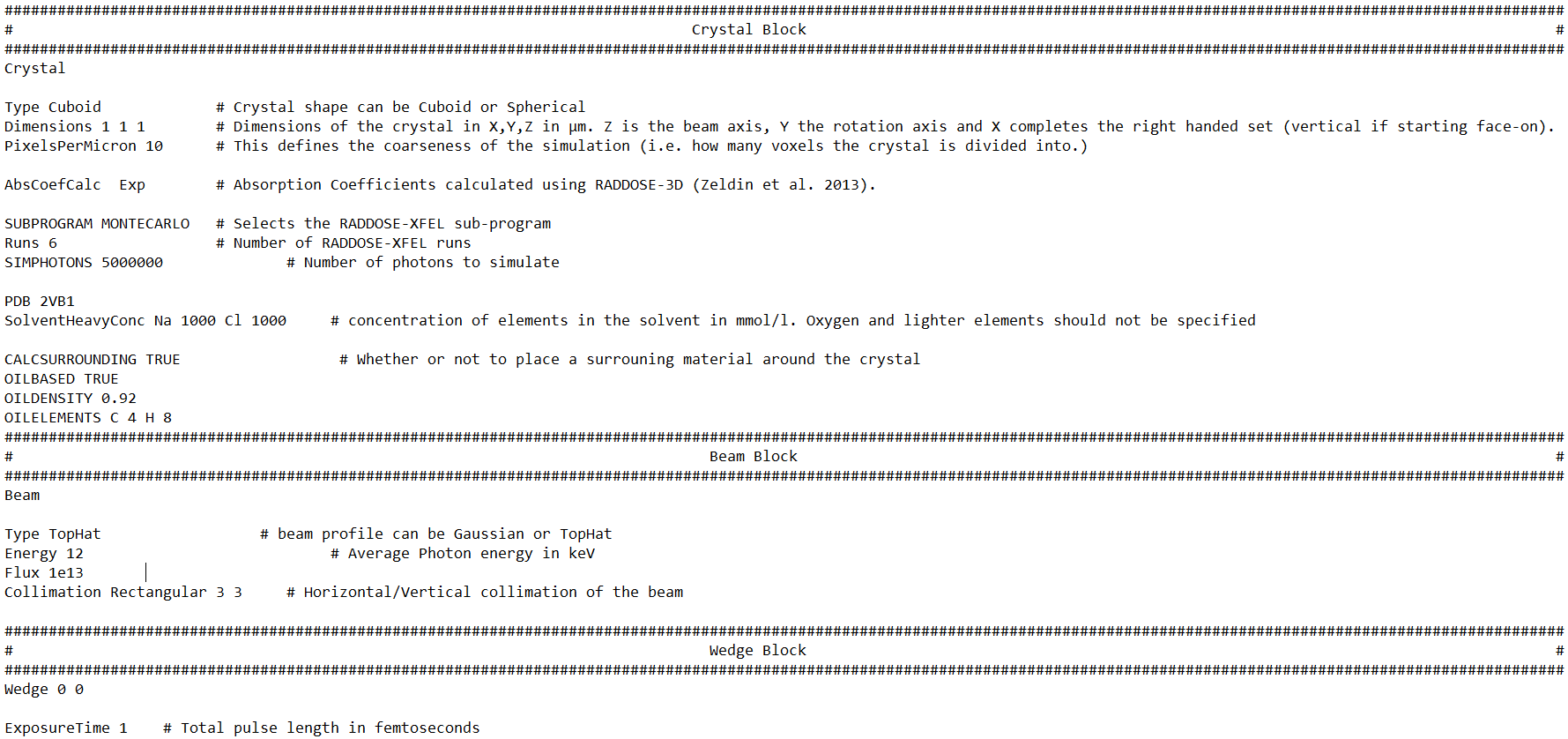


**Figure S1 – An example input file for the Monte Carlo simulations of dose absorbed by a protein crystal including photoelectron escape in RADDOSE-3D** and using an oil based surrounding material.
